# Feasibility and postoperative opioid sparing effect of an opioid-free anaesthesia in adult cardiac surgery: a retrospective study

**DOI:** 10.1186/s12871-021-01362-1

**Published:** 2021-06-03

**Authors:** Clément Aguerreche, Gaspard Cadier, Antoine Beurton, Julien Imbault, Sébastien Leuillet, Alain Remy, Cédrick Zaouter, Alexandre Ouattara

**Affiliations:** 1 CHU Bordeaux, Department of Anaesthesia and Critical Care, Magellan Medico-Surgical Centre, F-33000 Bordeaux, France; 2 Univ. Bordeaux, INSERM, UMR 1034, Biology of Cardiovascular Diseases, F-33600 Pessac, France; 3 Biofortis Mérieux NutriSciences, Saint-Herblain, France; 4 Department of Anaesthesia, University of Montreal, Centre Hospitalier de l’Universtié de Montréal, Montreal, Quebec Canada

**Keywords:** Opioid-free anaesthesia, Dexmedetomidine, Cardiac surgery, Morphine, Pain

## Abstract

**Background:**

No previous study investigated the dexmedetomidine-based opioid-free anesthesia (OFA) protocol in cardiac surgery. The main objective of this study was to evaluate the feasibility and the postoperative opioid-sparing effect of dexmedetomidine-based OFA in adult cardiac surgery patients.

**Methods:**

We conducted a single-centre and retrospective study including 80 patients above 18 years old who underwent on-pump cardiac surgery between November 2018 and February 2020. Patients were divided into two groups: OFA (lidocaine, ketamine, dexmedetomidine, MgSO4) or opioid-based anaesthesia (remifentanil and anti-hyperalgesic medications such as ketamine and/or MgSO4 and/or lidocaine at the discretion of the anesthesiologist). The primary endpoint was the total amount of opioid consumed in its equivalent of intravenous morphine during the first 48 postoperative hours. Secondary outcomes included perioperative hemodynamics, post-operative maximal pain at rest and during coughing and adverse outcomes. Data are expressed as median [interquartile range].

**Results:**

Patients in the OFA-group had a higher EuroSCORE II, with more diabetes, more dyslipidemia and more non-elective surgery but fewer smoking history. In the OFA group, the median loading dose of *dexmedetomidine* was 0.6 [0.4–0.6] μg.kg^− 1^ while the median maintenance dose was 0.11 μg.kg^− 1^.h^− 1^ [0.05–0.20]. In 10 (25%) patients, dexmedetomidine was discontinued for a drop of mean arterial pressure below 55 mmHg. The median total amount of opioid consumed in its equivalent of intravenous morphine during the first 48 postoperative hours was lower in the OFA group (15.0 mg [8.5–23.5] versus 30.0 mg [17.3–44.3], *p* < 0.001). While no differences were seen with rest pain (2.0 [0.0–3.0] versus 0.5 [0.0–5.0], *p* = 0.60), the maximal pain score during coughing was lower in OFA group (3.5 [2.0–5.0] versus 5.5 [3.0–7.0], *p* = 0.04). In OFA group the incidence of atrial fibrillation (18% versus 40%, *p* = 0.03) and non-invasive ventilation use (25% versus 48%, *p* = 0.04) were lower. The incidence of bradycardia and the intraoperative use of norepinephrine were similar between both groups.

**Conclusion:**

Dexmedetomidine-based OFA in cardiac surgery patients is feasible and could be associated with a lower postoperative morphine consumption and better postoperative outcomes. Further randomized studies are required to confirm these promising results and determine the optimal associations, dosages, and infusion protocols during cardiac surgery.

**Graphical abstract:**

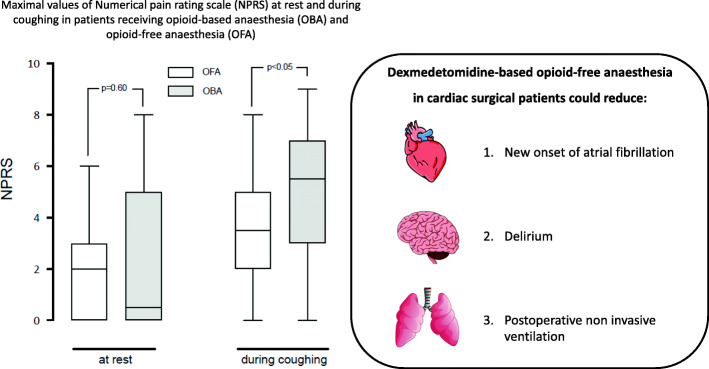

**Supplementary Information:**

The online version contains supplementary material available at 10.1186/s12871-021-01362-1.

## Introduction

As early as the 1990’s fast-track protocols have been implemented successfully lowering opioid doses and allowing rapid extubation after cardiac surgery using a balanced opioid anesthetic [1–3]. However, balanced opioid anesthesia may be responsible for hyperalgesia and acute tolerance which could lead to both an increase in opioid prescription [4] and postoperative chronic pain (nearly 20% 1 y after sternotomy) [5]. Recently nonopioid interventions including the intraoperative use of dexmedetomidine have been proposed to reduce opioid consumption during the perioperative period of cardiac surgery patients [6, 7]. Better pain control and lower opioid consumption seems to be crucial to enable the implementation of postoperative enhanced recovery elements such as early mobilization and early nutrition [6].

A milestone that could help reducing even further perioperative opioid consumption for cardiac surgery patients might be the integration of opioid-free anesthesia (OFA) protocol. In OFA for non-cardiac surgery, sympathetic nervous system control is obtained administrating a combination of several drugs studied the last 30 years such as intravenous lidocaine [8], ketamine [9], dexmedetomidine which is a highly selective alpha-2 agonist [10] and magnesium sulfate [11]. This multimodal analgesic approach has an important opioid sparing effect that has been shown to limit opioid-related side effects such as respiratory depression and, thus prolonged duration of mechanical ventilation, delirium, urinary retention, nausea, ileus and vomiting [12]. Few data on OFA in cardiac surgery demonstrating its feasibility are available [13, 14]. One retrospective study compared an OFA (protocol combining propofol-lidocaine-ketamine-dexamethasone) to an opioid-based anaesthesia (OBA) with sufentanil and regional anaesthesia [15]. Recent data suggest that dexmedetomidine added to a balanced anaesthesia protocol in cardiac surgical patients could reduce opioid consumption, postoperative pain and duration of mechanical ventilation [16, 17]. Interestingly, dexmedetomidine administration through this approach may also reduce postoperative myocardial injury, incidence of new onset of arrythmias and even postoperative mortality up to 1 year after cardiac surgery [18].

The main objective of the present retrospective study was to evaluate the feasibility and the postoperative opioid-sparing effect of dexmedetomidine-based OFA in adult cardiac surgery patients. We tested the hypothesis that dexmedetomidine-based OFA could significantly reduce morphine consumption during the first 48 h following on-pump cardiac surgery.

## Methods

### Patients

We performed a retrospective and single-centre study in a tertiary university hospital (Bordeaux, France) from November 2018 to February 2020.

The OFA protocol has been implemented in our institution from February 2018. After an initial period of several months, to guarantee good communication between every care provider and the compliance to the OFA protocol, we have started to recruit patients from November 2018. Thus, from November 2018 to February 2020 we have included retrospectively from our database 40 consecutive patients undergoing on-pump cardiac surgery and receiving an OFA [19, 20]. Data of these 40 OFA patients were compared to 40 other patients operated during the same period but receiving an OBA. During the study period (from November 2018 to February 2020), a total of 2108 consecutive patients underwent on-pump cardiac surgery. To prevent temporal bias, we took into account the temporal effect and obtained homogenous groups in time, sampling cases evenly in time across the recruitment period. Hence, 40 OBA patients were recruited and included in the analysis at the same pace. These 40 OBA patients were selected weekly (week recruitment period block), with a ratio of 1:1, from our database. OBA patients were selected identifying patients undergoing similar cardiac surgical procedure with equivalent cardiopulmonary bypass duration as patients in the OFA group. If for one week several patients responded to these criteria, we decided arbitrarily to include the first patient meeting such criteria in order to follow a chronological rational. Patients undergoing off-pump cardiac surgery and/or with pre-operative hemodynamic instability and/or with atrio-ventricular block grade 2 or 3 and/or hypersensitivity to opioids were excluded.

### Intraoperative management

Upon arrival in the operating room, routine monitoring (five lead-ECG, pulse oximeter, non-invasive arterial pressure) was instituted. A peripheral venous catheter and an arterial catheter were inserted under local anesthesia. After induction of anesthesia, hemodynamic monitoring was completed by inserting a triple lumen central venous catheter in the right internal jugular vein to infuse drugs and to monitor the central venous pressure. Anesthesia management is summarized in the supplementary material (additional files Table 1).

As previously published by our team [3], anesthesia in the OBA group was based on propofol and remifentanil both simultaneously administered via target-controlled infusion (TCI) using the Schnider’s [21] and the Minto’s [22] models, respectively. The induction of anesthesia was ensured with a target effect-site concentration of propofol between 2.0 and 4.0 μg.ml^− 1^ and remifentanil between 3.0 and 10.0 ng ml^− 1^. For the maintenance of anesthesia target effect-site concentrations of propofol and remifentanil were adapted to maintain bispectral index (Covidien, Boulder, CO, USA) value between 40 and 60 and to maintain a Mean Arterial Pressure (MAP) between 60 and 85 throughout all the surgical procedure, respectively. A 0.10–0.15 mg.kg^− 1^ bolus dose of morphine was given intravenously 30 min before the anticipated end of surgery for postoperative analgesia. In these patients, the intraoperative use of ketamine (IV bolus 0.3 mg.kg^− 1^ at the induction followed by continuous infusion 0.25 mg.kg^− 1^.h^− 1^) and /or lidocaine (1.5 mg.kg^− 1^ bolus followed by continuous infusion 1.5 mg.kg^− 1^.h^− 1^) and /or magnesium sulfate (3 g over 15 min at the induction) was left at the discretion of the attending anaesthetist.

In the OFA group, a pre-induction mixture of intravenous boluses of dexmedetomidine (0.3–0.6 μg.kg^− 1^ over 15 min), magnesium sulfate (3 g over 15 min), dexamethasone (0.1 mg.kg^− 1^) and lidocaine (1.5 mg.kg^− 1^) was given over 15 min. A bolus of ketamine (0.3 mg.kg^− 1^) was followed by continuous infusion (0.25 mg.kg^− 1^.h^− 1^), which was stopped at wound closure. Then, the anesthesia was induced by intravenous anaesthesia with TCI of propofol (2 to 4 μg. mL^− 1^). The maintenance of anesthesia was ensured by propofol administered via TCI using the Schnider’s target effect-site concentrations adapted to bispectral index values between 40 and 60. After the induction, a continuous infusion of dexmedetomidine (0.1 to 0.5 μg.kg^− 1^.h^− 1^) and lidocaine (1.5 mg.kg^− 1^.h^− 1^) were started. The continuous infusion of dexmedetomidine was adapted to MA*P* values. If MAP was below 55 mmHg during surgery, dexmedetomidine was completely discontinued. Conversely, if MAP was higher than 90 mmHg and BIS between the target values, dexmedetomidine was increased up to 0.5 μg.kg^− 1^.h^− 1^. When hypertension persisted despite these maximal doses, urapidil or nicardipine were given.

In both groups, no regional anesthesia was performed and the tracheal intubation was facilitated with neuromuscular blockade using cisatracurium bolus 0.15 mg.kg^− 1^ followed by a continuous infusion of 0.1 mg.kg^− 1^.h^− 1^ until aortic unclamping. Cardiopulmonary bypass (CPB) was conducted with a heart-lung machine (Stockert Sorin S5 Heart Lung, Milan, Italy) with a target blood flow of 2.4 L.min^− 1^.m^− 2^ or more if SvO_2_ was less than 70%. During CPB, the MAP was maintained above 55 mmHg increasing the pump flow rate, reducing propofol target if BIS was below 40, discontinuing dexmedetomidine infusion in the OFA group or decreasing remifentanil up to 2 ng. mL^− 1^ in the OBA group if BIS was above 40 or administrating vasoactive drugs (ephedrine, norepinephrine) if hypotension persisted. The CPB circuit was primed with 900 à 1200 ml of crystalloids (Plasma-Lyte®; Baxter, Lessines, Belgium) and 5000 UI of heparin. After systemic heparinization (300 UI.kg^− 1^) to reach an activated cephalin time above 420 s, median sternotomy was performed then aortic and right auricular cannulations were started. Perioperative hyperglycemia above 10 mmol. L^− 1^ was treated by intravenous insulin as elsewhere detailed [23]. Homologous red blood cell transfusions were guided by physiological parameters such as SvO_2_ and haemoglobin level when less than 7.5 g.dL^− 1^. Heparin was reversed with protamine at a 1:1 ratio.

In absence of counter-indication, all patients in each group received 30 min before the end of surgery, nefopam (IV bolus 20 mg followed by an infusion of 100 mg over 24 h) and paracetamol (1 g followed by 1 g every 6 h). Remifentanil, ketamine, lidocaine and dexmedetomidine were stopped at the end of the surgical dressing. Only propofol was continued in all patients during the intensive care unit (ICU) transfer.

### ICU management

Upon arrival in ICU, postoperative sedation was ensured with a continuous propofol infusion. Propofol infusion was stopped and patients extubated once blood loss was considered acceptable (less than 1 ml. kg^− 1^.h^− 1^), chest x-ray ruled out complications, a hemodynamic stability, a normothermia and no residual neuromuscular blockade (train-of-four ratio measured at the adductor pollicis muscle > 90%) were obtained. The scheduled blood tests on admission to the ICU included arterial blood gas measurements and hypersensitivity cardiac troponin I (hs-cTnI) between 12 and 24 h after surgery. Pain was assessed as early as possible after the ICU arrival using a numerical pain rating scale (NPRS). Initial analgesia consisted of morphine titration with a bolus of 3 mg if NPRS was greater than 3. Then, morphine patient-controlled analgesia was started as follow: 1 mg bolus, refractory period of 7 min, maximum dose of 20 mg every 4 h without continuous infusion. Then, pain was assessed at least every 2 h by nurses during the ICU stay using the NPRS. Intravenous rescue analgesia was given if NPRS score was > 3 and was left to the discretion of the attending physician and included ketoprofen (50–100 mg every 8 h) and /or tramadol (50–100 mg every 6 h) and/or ketamine boluses (10–20 mg) and/or oral oxycodone (5–10 mg maximum 6 per day). Non-invasive ventilation indications were high-risk patients (obesity, chronic obstructive pulmonary disease), atelectasis, hypoxemia, hypercapnia, obstructive sleep apnea without personal equipment and acute respiratory failure. Patients were discharged from ICU at the discretion of their attending physician. The following variables were continuously recorded in the institutional database [19, 20]: age, gender, body weight, height, personal medical history and medicines, Euro-SCORE II, type of cardiac surgery, the preoperative left ventricular ejection fraction, the duration of CPB, intraoperative blood transfusion, norepinephrine, dobutamine or milrinone, antihypertensive agent (nicardipine, urapidil), atropine, creatinine value, time to extubation (hours), arrythmias or conduction blockade and any other occurrence of complications during the ICU or in-hospital stay, and the length of stay (LOS) in the ICU and hospital.

### Outcomes

The primary endpoint was the total amount of opioid consumed in its equivalent of intravenous morphine during the first 48 postoperative hours and included intravenous morphine given at the end of surgery, the titration dose, the morphine administered via a patient-controlled analgesia, the dose of oral oxycodone prescribed postoperatively on the surgical ward with the following conversion ratios: oral morphine/oxycodone 2:1 and oral morphine/IV morphine 1:3 and the tramadol dose with the following conversion ratio: tramadol/IV morphine 1:15 [24]. The secondary endpoints were the intraoperative fluid expansion, intraoperative vasoactive agent administration, median maximal values of NPRS at rest and during coughing within the first post-operative 48-h, analgesia rescue requirement and the rate of non-invasive ventilation support, new onset of atrial fibrillation, and postoperative delirium defined as episode of confusion in nursing or medical observation. Secondary outcomes included also postoperative stroke and/or seizure, the incidence acute kidney injury defined as a Kidney Disease: Improving Global Outcomes stage 2 or 3*,* the postoperative level of hs-cTnI, ICU and hospital length of stay, and the hospital mortality rate. All data were collected from our institutional informatic database by a physician who was not involved in the care of the study patients.

### Statistical analysis

The Shapiro-Wilks normality test was used to assess the normality of quantitative outcomes. In case of normality, quantitative variables were expressed as mean (SD) and a Student test was used to compare the OBA group with the OFA groups. If non normality was assumed, these variables were presented as interquartile range (IQR) and were compared using a Mann-Whitney-Wilcoxon test. Categorical outcomes were expressed as number (percentage) and were compared using a Chi-Square test or Fischer’s Exact tests (when the expected values in one of the cells of the contingency table was less than 5). Statistical analyses were conducted using GraphPad Prism version 8.4.3 (GraphPad Software, San Diego, California, USA). For all the statistical tests, a 0.05 significance level was used to claim a statistically significant effect and all reported *p* values are from 2-sided tests. The sample size was determined from a preliminary retrospective analysis including 18 patients treated using an OBA protocol but no included in the final analysis. In these patients, the mean dose of morphine sulfate equivalents consumed during the first 48 postoperative hours was 21 ± 8 mg. Considering a 30% decrease in patients treated with an OFA protocol as clinically relevant, a sample size of 35 patients per group provided 90% power with a two-sided type I error of 0.05 to show this difference. Taking into account an anticipated loss-to-follow-up rate of 10%, a total of 40 patients per group was planned.

### Ethics

This retrospective observational study was conducted in accordance with the ethical standards of the declaration of Helsinki and relevant guidelines and regulations. In accordance with French law [25], this study was approved by our ethics committee (Comité d’Ethique du Centre Hospitalier Universitaire de Bordeaux-Groupe Publication) on August 13, 2020 (reference number GP – CE2020–33 by Chair Dr. Thibaud Haaser). The design of the study complies with the general data protection regulation n ° 2016/679 / EU of April 27, 2016 and falls within the framework of article 65–2 of the Data Protection Act n ° 78–17 of January 6, 1978 modified 2018. Consequently, it does not require a declaration to the national supervisory authority. Because the current study was a retrospective observational trial with patients treated according to our hospital standard of care, our ethics committee (Comité d’Ethique du Centre Hospitalier Universiataire de Bordeaux-Groupe Publication) granted an authorisation to waive written informed consent from patients. In addition, the other conditions relating to the right to privacy and the protection of personal health data were approved by the data protection officer and the study was recorded in the processing register under the reference CHUBX2020RE0260. All data were collected and analyzed confidentially assigning an identification number to each patient.

## Results

### Characteristics of the population

During the study period, 80 patients were included, and were divided in two groups: the OFA group (*n* = 40) and the OBA group (*n* = 40). In our study, patients in the OFA-group were sicker and underwent more often non-elective surgery (Table 1). During the recruitment period matching between the groups was not possible. However, patients’ inclusion in the study occurred during the same time frame and pace. The surgical procedure and length of surgery were similar (Table 2). The incidence of preoperative chronic pain (7% vs 4%, *p* = 0.33) or opioid consumption (3% vs 1%, *p* = 0.30) were similar between the OFA-group and the OBA-group.

**Table 1:** Baseline characteristics of patients receiving opioid-free anaesthesia (OFA) or opioid-based anaesthesia (OBA)

	All patients (*n* = 80)	OFA (*n* = 40)	OBA (*n* = 40)	*P*-value
Age, years	71 [64–75]	71 [63–74]	71 [67–75]	0.81
Male gender	56 (70)	25 (63)	31 (78)	0.14
Body mass index, kg.m^−2^	25.9 [24.2–30.0]	26.0 [24.3–30.5]	25.8 [24.2–28.6]	0.92
EuroSCORE II, %	3.0 [1.8–6.9]	4.7 [2.1–9.1]	2.0 [1.4–3.9]	< 0.001
Medical history				
Redo surgery	9 (11)	4 (10)	5 (13)	> 0.99
Active endocarditis	16 (20)	9 (23)	7 (18)	0.58
COPD	10 (13)	6 (15)	4 (10)	0.50
Obstructive sleep apnea	11 (14)	5 (13)	6 (15)	0.75
Smoking history	14 (18)	3 (8)	11 (28)	0.04
Hypertension	63 (79)	32 (80)	31 (78)	0.78
Dyslipidemia	41 (51)	25 (63)	16 (40)	0.04
Diabetes	18 (23)	13 (33)	5 (13)	0.03
Atrial fibrillation	12 (15)	5 (13)	7 (18)	0.53
History of stroke	8 (10)	3 (8)	5 (13)	0.71
Apfel score	2 [2–2]	2 [2–3]	2 [2–2]	< 0.01
LVEF, %	60 [50–60]	50 [50–60]	60 [55–65]	< 0.001
Creatinine clearance, mL.min^−1^	78 [62–96]	78 [63–94]	77 [62–101]	0.79
Non elective surgery	25 (31)	20 (50)	5 (13)	< 0.01
Preoperative medication				
Beta-blockers	36 (45)	15 (38)	21 (51)	0.18
Calcium channel blocker	23 (29)	13 (33)	10 (25)	0.46
Antiplatelet therapy*	35 (44)	19 (48)	16 (40)	0.18
ACEI	43 (54)	22 (55)	21 (53)	0.82
Statins	28 (35)	17 (43)	11 (28)	0.16

Data are presented as median [Interquartile range] or number (%) of patients. *EuroSCORE II* European System for Cardiac Operative Risk Evaluation II, *COPD* Chronic Obstructive Pulmonary Disease, *LVEF* Left Ventricular Ejection Fraction, * aspirin and/or clopidogrel, *ACEI* Angiotensin-conversing-enzyme inhibitors. *P* value refers to comparison between OFA and OBA groups

**Table 2:** Intraoperative characteristics of patients receiving opioid-free anaesthesia (OFA) or opioid-based anaesthesia (OBA)

	All patients (*n* = 80)	OFA (*n* = 40)	OBA (*n* = 40)	*P*-value
Anaesthesia time, min	270 [210–316]	293 [222–321]	258 [210–306]	0.38
Maximal target effect-site concentration of propofol for induction, μg.mL^−1^	2.5 [2.0-3.0]	2.0 [1.0–2.0]	3.0 [3.0–4.0]	< 0.01
Lowest heart rate before CPB	55 [47-60]	55 [48–60]	55 [45–64]	0.74
CPB time, min	80 [66–103]	82 [68–103]	79 [63–105]	0.81
Type of surgery				
Valvular surgery	31 (39)	14 (35)	17 (43)	0.49
CABG	22 (28)	14 (35)	8 (20)	0.13
Combined	25 (31)	11 (28)	14 (35)	0.47
Ascending aorta	2 (3)	1(3)	1 (3)	> 0.99
Intraoperative fluid, ml.kg^−1^	15 [9–23]	14 [8–23]	17 [10–22]	0.43
RBC transfusion	25 (31)	11 (28)	14 (35)	0.47
Urapidil use	3 (4)	3 (8)	0 (0)	0.08
Atropine use	2 (3)	0 (0)	2 (5)	0.15
Vasopressors requirements				
Norepinephrine (IV infusion)	40 (50)	17 (43)	23 (58)	0.18
Norepinephrine (boluses)	29 (36)	17 (43)	12 (30)	0.24
Ephedrine	9 (11)	1 (3)	8 (20)	0.03
Inotropes use*	9 (11)	4 (10)	5 (13)	1.00

Data are presented as median [interquartile range] or number (%) of subjects. CPB: cardiopulmonary bypass; *CABG* coronary artery bypass graft, *RBC* red blood cell; *: dobutamine and/or milrinone. *P* value refers to comparison between OFA and OBA groups

### Intraoperative period

In the OFA group, the median loading dose of dexmedetomidine received before induction of anesthesia was 0.6 [0.4–0.6] μg.kg^− 1^ while the median maintenance dose was 0.11 μg.kg^− 1^.h^− 1^ [0.05–0.20]. In 10 (25%) patients, dexmedetomidine was discontinued for a drop of mean arterial pressure below 55 mmHg. The median maximal target effect-site concentration of remifentanil for the induction of anesthesia was 4.0 [3.0–4.0] ng.ml^*− 1*^*.* A larger number of patients in the OBA-group required intraoperatively ephedrine (Table 2).

### Perioperative analgesia and outcomes

**Fig. 1:**
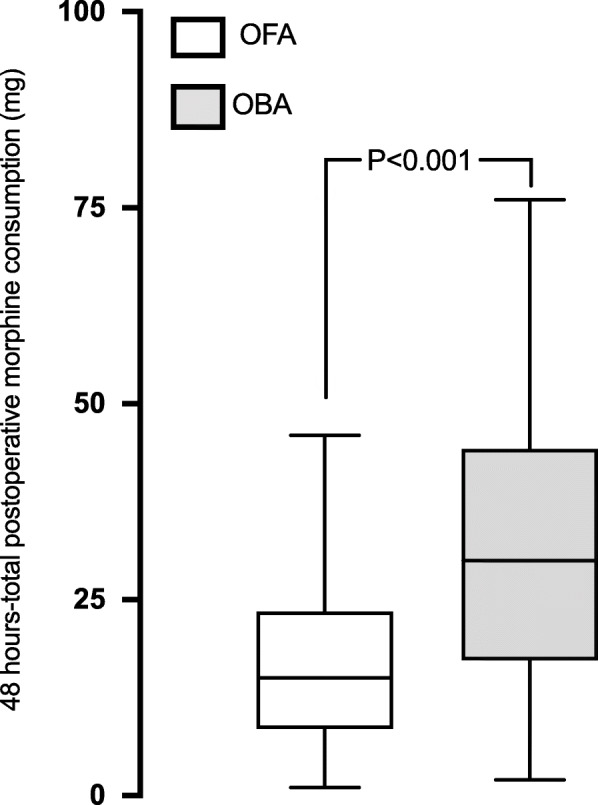
Box-plot showing the total postoperative morphine consumption during the first 48 hours in the OFA and OBA groups. The line inside the box represents the median, box edges represent 25th and 75th percentile and the whiskers represent the minimum and maximum values

A large proportion of patients received paracetamol and nefopam with no difference between groups. A comparable proportion of patients received a morphine titration (58% versus 70%, *p* = 0.24) and received rescue analgesia during the first 48 postoperative hours in both groups (Table 3). The primary outcome defined as the total amount of opioid consumed in its equivalent of intravenous morphine during the first 48 postoperative hours was significantly lower in the OFA-group compared to the OBA group (15.0 mg [IQR 8.5–23.5] versus 30.0 mg [IQR 17.3–44.3], *p* < 0.001) (Fig. 1). Maximal pain scores at rest were similar between the two groups (2.0 [0.0–3.0] in the OFA group versus 0.5 [0.0–5.0] in the OBA group, *p* = 0.60) but was lower in the OFA-group during coughing (3.5 [2.0–5.0] vs 5.5 [3.0–7.0], *p* = 0.04). No patient developed neither stroke nor seizure postoperatively. Patients in the OFA group presented a lower incidence of atrial fibrillation and required less frequently non-invasive ventilation (Table 4). We could observe a trend toward a reduction of new onset of postoperative delirium in patients receiving OFA but it did not reach a statistical significance. One patient in the OFA-group died (after a month due to a cessation of care because of a metastatic cancer discovered postoperatively during its ICU stay).

**Table 3:** Peri-operative analgesia in patients receiving opioid-free anaesthesia (OFA) and opioid-based anaesthesia (OBA)

	All patients (*n* = 80)	OFA (*n* = 40)	OBA (*n* = 40)	*P*-value
Intraoperative analgesia				
Paracetamol	79 (99)	39 (98)	40 (100)	> 0.99
Nefopam	72 (90)	38 (95)	34 (85)	0.26
Ketoprofen	44 (55)	32 (80)	12 (30)	< 0.01
Tramadol	2 (3)	2 (5)	0 (0)	0.49
Morphine	33 (41)	1 (3)	32 (80)	< 0.01
Ketamine, *n* (%)	76 (95)	40 (100)	36 (90)	0.12
Ketamine dose, mg	75 [50–100]	90 [75–100]	50 [30–70]	< 0.001
Lidocaine, *n* (%)	50 (63)	40 (100)	10 (25)	< 0.01
MgSO_4_^2−^	48 (60)	40 (100)	8 (20)	< 0.01
Morphine titration	51 (64)	23 (58)	28 (70)	0.24
Rescue analgesia during first 48 h				
Ketoprofen use	30 (38)	18 (45)	12 (30)	0.17
Ketoprofen dose, mg	100 [50–150]	100 [75–200]	100 [50–100]	0.18
Tramadol use	7 (9)	5 (13)	2 (5)	0.43
Tramadol dose, mg	200 [125–425]	200 [100–400]	325 [125–425]	0.57
Oxycodone oral	23 (29)	9 (23)	14 (35)	0.22
Ketamine use	8 (10)	2 (5)	6 (15)	0.26

Data are presented as median [Interquartile range] or number (%) of patients. P value refers to comparison between OFA and OBA groups

**Table 4:** Postoperative outcomes patients receiving opioid-free anaesthesia (OFA) and opioid-based anaesthesia (OBA)

	All patients (*n* = 80)	OFA (*n* = 40)	OBA (*n* = 40)	*P*-value
PaO_2_/FiO_2_ ratio at ICU arrival	309 ± 99	327 ± 112	291 ± 81	0.10
Lowest PaO_2_/FiO_2_ ratio during first 48 h	238 [188–278]	244 [189–288]	226 [186–273]	0.49
Noninvasive ventilation use	29 (36)	10 (25)	19 (48)	0.04
Time to extubation, hours	3 [2–5]	2 [2–3]	4 [2–7]	< 0.01
Reintubation	4 (5)	2 (5)	2 (5)	1.00
Vomiting and/or nausea	8 (10)	3 (8)	5 (13)	0.71
Surgical re-exploration	7 (9)	1 (3)	6 (15)	0.11
Need for vasopressors ≥6 h	23 (29)	13 (33)	10 (25)	0.46
Need for inotropes ≥6 h	9 (11)	4 (10)	5 (13)	> 0.99
New onset of atrial fibrillation	23 (29)	7 (18)	16 (40)	0.03
Ventricular arrhythmias*	5(6)	2 (5)	3 (8)	> 0.99
Atrioventricular block^£^	1 (1)	0	1 (3)	> 0.99
Delirium	8 (10)	1 (3)	7 (18)	0.06
ARF (KDIGO stage 2 or 3)	5 (6)	1 (3)	4 (10)	0.36
Hs-cTnI at 12 h, ng. L^−1^	1700 [918–4925]	1700 [900–3500]	1700 [1000–8200]	0.44
ICU length of stay, hours	48 [41–73]	47 [35–84]	49 [42–73]	0.55
Hospital length of stay, days	12 [9–15]	11 [10–15]	13 [9–15]	0.48

Data are presented as median [Interquartile range] or mean ± standard deviation or number (%) of patients. *ICU* intensive care unit, * ventricular or fibrillation, ^*£*^ high-grade atrioventricular block requiring pacemaker implantation, *ARF* acute renal failure, *KDIGO* Kidney Disease Improving Global Outcomes, *Hs-cTnI* high-sensitivity cardiac troponin I. *P* value refers to comparison between OFA and OBA groups

## Discussion

The major findings of our study are that dexmedetomidine-based OFA: 1) appears to be feasible, 2) has a statistically significant opioid sparing effect without obviously altering pain relief and 3) could be associated with better postoperative outcomes including less new onset of atrial fibrillation, a lower rate of postoperative need for non-invasive ventilation and perhaps less incidence of postoperative delirium.

### Feasibility

Only one previous study evaluated the feasibility of OFA based on lidocaine and ketamine in cardiac surgery [15]. Despite a higher intra operative use of esmolol and urapidil, these authors reported that OFA reduces significantly postoperative morphine consumption [15]. A large opioid sparing effect was also observed in patients receiving an OFA protocol [15]. However, the OFA protocol used in our patients was substantially different and was based on a pre-induction mixture of intravenous infusion of dexmedetomidine, magnesium sulfate and lidocaine. Dexmedetomidine has been well studied as an adjunct in balanced anaesthesia for cardiac surgery but no previous study has ever evaluated the benefit of dexmedetomidine-based OFA strategy [16, 17]. A safety and an efficient analgesic effect of dexmedetomidine in cardiothoracic surgery has been previously reported [16]. The hemodynamic effects of lidocaine have been previously investigated in cardiac surgical patients [26]. A 1.5 mg.kg^− 1^ intravenous bolus of lidocaine effectively limits increase in arterial pressure during aortic canulation [26]. Concerning the use of ketamine, its sympathomimetic effect could potentially lead to an increase in myocardial oxygen consumption [27]. Even if our OFA patients received a larger intraoperative amount of ketamine, no significant difference in postoperative hs-cTnI level was observed. Magnesium sulfate has a vasodilator effect and could potentiate the hypotensive effects of propofol [28]. For this reason, we have administered magnesium intravenously slowly over a period of 15 min. Because magnesium sulfate reduces intraoperative hemodynamic variability, some authors proposed its intraoperative use to control sympathetic response to surgery during OFA [29]. Moreover, magnesium sulfate significantly reduces requirement for anesthetic drugs and may potentiate neuromuscular blockade in cardiac surgery patients [30, 31]. Additionally, a high incidence of postoperative residual curarisation in patients undergoing long duration non-cardiac surgery intervention and for whom the block is not antagonized [32]. In accordance with our daily clinical practice, absence of postoperative residual curarization was systematically eliminated before to stop propofol infusion and perform tracheal extubation. Our findings suggest that dexmedetomidine-based OFA is feasible. Although, dexmedetomidine has been discontinued in 10 (25%) patients, the intraoperative use of vasopressors was comparable between groups. This finding confirms results obtained from a meta-analysis conducted in non-cardiac surgery [33]. In addition, we did not observe a higher incidence of postoperative vasoplegia in the OFA-group. Previous studies conducted in cardiac surgical patients reported an increased risk of bradycardia with dexmedetomidine. However, it should be pointed out that in these studies dexmedetomidine was used as an adjunct to an opioid based-anaesthesia strategy.

### Opioid sparing effect and analgesia

Dexmedetomidine analgesic and opioid-sparing effects are dose-dependent and trigger at spinal cord sites as well as through non-spinal mechanisms [34]. It has been suggested that alpha-2 agonist receptors activation, inhibition of the C and A delta fibres signals conduction, and the local release of encephalin are the underlying non-spinal mechanisms of dexmedetomidine to provide anti-nociception effects [35]. Grant et al. [7] showed that with an enhanced recovery program for cardiac surgery, the intraoperative opioid sparing effect was greater when preoperative acetaminophen, gabapentin, intraoperative ketamine and dexmedetomidine infusions, and regional analgesia (via a serratus anterior plane block) were combined. In the analysis of each individual intervention effect, dexmedetomidine was the molecule associated with the best intra operative opioid sparing effect [7]. For non-cardiac surgery, lidocaine combined with dexmedetomidine infusion significantly improve postoperative pain and lower opioid-related side effects such as bowel function or nausea [36, 37]. Ketamine via its anti N-methyl-D-aspartate (NMDA) effect reduces postoperative hyperalgesia, provides analgesia, hypnosis and amnesia [38]. Ketamine as an analgesic adjunct reduces opioid consumption after cardiac surgery and reduces variability of blood pressure [29, 39]. Some studies suggest an anti-inflammatory effect attenuating the inflammatory response to cardiopulmonary bypass and a delirium preventing effect [40]. By its antagonistic effect of NMDA receptor, magnesium sulfate minimizes postoperative pain, reduces requirement for analgesics and thus may have opioid sparing effect [41, 42]. Maximal NPRS scores at rest were similar between the two groups but NPRS scores were lower during coughing in the OFA-group in accordance with a study conducted in thoracic surgery [43]. Our present data seem to indicate that an OFA protocol designed for cardiac surgery could further decrease perioperative opioid consumption compared to the OBA group that received a multimodal analgesia using opioid intraoperatively. The present study shows that OFA could lower by half the postoperative opioid consumption. A such reduction should be considered as clinically relevant regarding to most of the patients undergoing cardiac surgery are elderly and to when a cardiac ERAS program is sought to be implemented [6].

### Secondary outcomes

The shorter extubation time in patients receiving OFA may appear to be surprising. No previous study reported similar result when dexmedetomidine was compared to remifentanil. However, the fact that surgical re-exploration for excessive bleeding was 5 times more frequent in the opioid anesthesia group must have confounded significantly the length of mechanical ventilation.

The OFA protocol was associated with better relevant outcomes in the post-operative course such as new onset of atrial fibrillation, a common event after cardiac surgery source of great morbidity and mortality [44]. Magnesium sulfate can have a preventive anti-arrhythmic effect on AF [45]. Dexmedetomidine can also have a protective effect in on-pump CABG [18] by decreasing myocardial ischemia-reperfusion and improving myocardium perfusion, anti-inflammatory [46, 47], sympatholytic and parasympathomimetic effect [48]. Lidocaine has anti-inflammatory effect, increases the cardioprotective effect of cardioplegia and decreases the risk of arrythmias but only of ventricular fibrillation [49]. Nevertheless, the incidence of ventricular arrythmias was too low in our study to show any benefit.

Interestingly, patients receiving OFA trend to present less postoperative delirium. Even if this difference was not significant, this beneficial effect may be explained by the opioid sparing effect observed and/or intrinsic effect of dexmedetomidine [50]. Moreover, intraoperative use of lidocaine could be protective against postoperative cognitive dysfunction modulating the cerebral inflammation secondary to cardiopulmonary bypass [51].

Our findings suggest the synergistic effect and multiple action site of the drugs used in the OFA-group could improve post-operative pain lowering the incidence of the side effects of each drug. Moreover, the additive anti-inflammatory effects of each drug may lower the most frequent postoperative complications.

### Limitations

The present study had several limitations, and the following points must be considered in the assessment of the clinical relevance of our study. First, our work is a single-centre retrospective observational study which did not control for any variables between the groups. Consequently, several differences between the two groups could be observed in baseline patients’ characteristics, mostly EuroSCORE II, non-elective surgery, LVEF, diabetes, dyslipidaemia and Apfel score; but all disadvantaging the OFA-group. Thus, in light of these drawbacks it could be claimed that a dexmedetomidine-based OFA for cardiac surgery could offer a good hemodynamic stability even in more fragile cardiac surgery patients. Second, at the moment of the study, OFA was an anesthetic protocol starting to be implemented within our department of anesthesia. Consequently, the thought process behind one patient being in the OFA group versus the OBA group was mainly conditioned by the attending anesthesiologist. This aspect could highlight the benefit of a clinically well conducted OFA-protocol. This also explains the long period of time necessary to obtain this relatively low number of patients and limits its external validity. Third, in the OBA group the intraoperative use of anti-hyperalgesic medications such as ketamine and/or magnesium sulfate and/or lidocaine was left at the discretion of the attending anesthesiologist. It would have been easier to compare the OFA and the OBA group if all patients in the OBA group have received these anti-hyperalgesic medications. Fourth, remifentanil use for the opioid-based approach may make this medication a poor choice when designing a trial that compares an opioid-free to an opioid based approach because of the potential for this medication could lead to postoperative hyperalgesia [52]. Fifth, ketamine boluses used for postoperative analgesic management could not be converted to a morphine equivalent dose, thus this analgesic administration was not taken into account for the total morphine consumption. Finally, because all of the multimodal agents being simultaneously administered it appears difficult to clearly determine the specific role of dexmedetomidine acting as an opioid-sparing agent. However, the present study offers central clinical hints on the potential of a dexmedetomidine-based OFA protocol designed for cardiac surgery patients. Nevertheless, only controlled prospective randomized studies will confirm the present results. Further studies are needed to determine the optimal associations, dosages, and infusion protocols for cardiac surgery patients.

## Conclusion

Our study strongly suggests that dexmedetomidine-based OFA in adult cardiac surgery is feasible and provides intraoperative hemodynamic stability. A such anaesthetic approach is responsible for postoperative opioid sparing effect and might have some clinically relevant benefits to improve outcomes.

## Supplementary Information

**Additional file 1: Table S1.** Anesthesia management in OFA (opioid free anesthesia (OFA) and opioid based anesthesia (OBA) groups

## Data Availability

All relevant data was presented within the manuscript and the datasets used and/or analyzed during the current study are available from the corresponding author on reasonable request.

## References

[1] D’Attellis N, Nicolas-Robin A, Delayance S (1997). Early extubation after mitral valve surgery: a target-controlled infusion of propofol and low-dose sufentanil. J Cardiothorac Vasc Anesth.

[2] Cheng DC, Newman MF, Duke P, Wong DT, Finegan B, Howie M, Fitch J, Bowdle TA, Hogue C, Hillel Z, Pierce E, Bukenya D (2001). The efficacy and resource utilization of remifentanil and fentanyl in fast-track coronary artery bypass graft surgery: a prospective randomized, double-blinded controlled, multi-center trial. Anesth Analg.

[3] Ouattara A, Boccara G, Lemaire S, Ko¨ckler U, Landi M, Vaissier E, Léger P, Coriat P (2003). Target-controlled infusion of propofol and remifentanil in cardiac anaesthesia: influence of age on predicted effect-site concentrations. Br J Anaesth.

[4] Scholl L, Seth P, Kariisa M (2018). Drug and opioid-involved overdose deaths — United States, 2013–2017. MMWR Morb Mortal Wkly Rep.

[5] van Gulik L, Ahlers SJGM, van de Garde EMW, Bruins P, van Boven WJ, Tibboel D, van Dongen EPA, Knibbe CAJ (2012). Remifentanil during cardiac surgery is associated with chronic thoracic pain 1 yr after sternotomy. Br J Anaesth.

[6] Zaouter C, Damphousse R, Moore A, Stevens LM, Gauthier A, Carrier FM. Elements not graded in the cardiac enhanced recovery after surgery guidelines might improve postoperative outcome: a comprehensive narrative review. J Cardiothorac Vasc Anesth. 2021. 10.1053/j.jvca.2021.01.035. 10.1053/j.jvca.2021.01.035 33589344

[7] Grant MC, Isada T, Ruzankin P, Gottschalk A, Whitman G, Lawton JS, Dodd-o J, Barodka V (2020). Opioid-sparing cardiac anesthesia: secondary analysis of an enhanced recovery program for cardiac surgery. Anesth Analg.

[8] Kranke P, Jokinen J, Pace NL, et al. Continuous intravenous perioperative lidocaine infusion for postoperative pain and recovery. Cochrane Database Syst Rev. 2015:CD009642. 10.1002/14651858.CD009642.pub2 26184397

[9] Bell RF, Dahl JB, Moore RA, et al. Perioperative ketamine for acute postoperative pain. Cochrane Database Syst Rev. 2006:CD004603. 10.1002/14651858.CD004603.pub2 16437490

[10] Blaudszun G, Lysakowski C, Elia N, Tramèr MR (2012). Effect of perioperative systemic α2 agonists on postoperative morphine consumption and pain intensity: systematic review and meta-analysis of randomized controlled trials. Anesthesiology.

[11] Cavalcanti IL, De Lima FLT, Da Silva MJS (2019). Use profile of magnesium sulfate in anesthesia in Brazil. Front Pharmacol.

[12] Aziz NA, Chue MC, Yong CY (2011). Efficacy and safety of dexmedetomidine versus morphine in post-operative cardiac surgery patients. Int J Clin Pharm.

[13] Landry E, Burns S, Pelletier MP, Muehlschlegel JD (2019). A successful opioid-free anesthetic in a patient undergoing cardiac surgery. J Cardiothorac Vasc Anesth.

[14] Cardinale JP, Gilly G (2018). Opiate-free tricuspid valve replacement: case report. Semin Cardiothorac Vasc Anesth.

[15] Guinot PG, Spitz A, Berthoud V, et al. Effect of opioid-free anaesthesia on post-operative period in cardiac surgery: a retrospective matched case-control study. BMC Anesthesiol. 2019;19:136. 10.1186/s12871-019-0802-y. 10.1186/s12871-019-0802-y PMC6668113 31366330

[16] Habibi V, Kiabi FH, Sharifi H (2018). The effect of dexmedetomidine on the acute pain after cardiothoracic surgeries: a systematic review. Braz J Cardiovasc Surg.

[17] Lin YY, He B, Chen J, Wang Z (2012). Can dexmedetomidine be a safe and efficacious sedative agent in post-cardiac surgery patients? A meta-analysis. Crit Care.

[18] Ren J, Zhang H, Huang L (2013). Protective effect of dexmedetomidine in coronary artery bypass grafting surgery. Exp Ther Med.

[19] Duval B, Besnard T, Mion S, Leuillet S, Jecker O, Labrousse L, Rémy A, Zaouter C, Ouattara A (2019). Intraoperative changes in blood lactate levels are associated with worse short-term outcomes after cardiac surgery with cardiopulmonary bypass. Perfusion.

[20] Mion S, Duval B, Besnard T, Darné B, Mouton C, Jecker O, Labrousse L, Remy A, Zaouter C, Ouattara A (2020). U-shaped relationship between pre-operative plasma fibrinogen levels and severe peri-operative bleeding in cardiac surgery: a report from the perioperative events aSSessment in adult cardiac surgery (PESSAC) registry. Eur J Anaesthesiol.

[21] Schnider TW, Minto CF, Shafer SL, Gambus PL, Andresen C, Goodale DB, Youngs EJ (1999). The influence of age on propofol pharmacodynamics. Anesthesiology.

[22] Minto CF, Schnider TW, Egan TD, Youngs E, Lemmens HJ, Gambus PL, Billard V, Hoke JF, Moore KH, Hermann DJ, Muir KT, Mandema JW, Shafer SL (1997). Influence of age and gender on the pharmacokinetics and pharmacodynamics of remifentanil. I. Model development. Anesthesiology.

[23] Cheisson G, Jacqueminet S, Cosson E, Ichai C, Leguerrier AM, Nicolescu-Catargi B, Ouattara A, Tauveron I, Valensi P, Benhamou D (2018). Perioperative management of adult diabetic patients. Intraoperative period. Anaesth Crit Care Pain Med.

[24] Nielsen S, Degenhardt L, Hoban B, Gisev N (2016). A synthesis of oral morphine equivalents (OME) for opioid utilisation studies. Pharmacoepidemiol Drug Saf.

[25] Toulouse E, Lafont B, Granier S, Mcgurk G, Bazin JE (2020). French legal approach to patient consent in clinical research. Anaesth Crit Care Pain Med.

[26] Totonchi Z, Salajegheh S, Mohaghegh MR, Kiaei MM, Shirvani M, Ghorbanlo M (2017). Hemodynamic effect of intravenous lidocaine during aortic cannulation in cardiac surgery. Interv Med Appl Sci.

[27] Patschke D, Brückner JB (1975). Gethmann JW, et al: [the effect of ketamine on haemodynamics and myocardial oxygen consumption in anaesthetized dogs (author’s transl)]. Prakt Anaesth.

[28] Dubé L, Granry JC (2003). The therapeutic use of magnesium in anesthesiology, intensive care and emergency medicine: a review. Can J Anesth.

[29] Forget P, Cata J (2017). Stable anesthesia with alternative to opioids: are ketamine and magnesium helpful in stabilizing hemodynamics during surgery? A systematic review and meta-analyses of randomized controlled trials. Best Pract Res Clin Anaesthesiol.

[30] Rodríguez-Rubio L, Nava E, del Pozo JSG, Jordán J (2017). Influence of the perioperative administration of magnesium sulfate on the total dose of anesthetics during general anesthesia. A systematic review and meta-analysis. J Clin Anesth.

[31] Pinard AM, Donati F, Martineau R, Denault AY, Taillefer J, Carrier M (2003). Magnesium potentiates neuromuscular blockade with cisatracurium during cardiac surgery. Can J Anesth.

[32] Cammu G, De Baerdemaeker L, Den Blauwen N, De Mey JC, Struys M, Mortier E (2002). Postoperative residual curarization with cisatracurium and rocuronium infusions. Eur J Anaesthesiol.

[33] Grape S, Kirkham KR, Frauenknecht J, Albrecht E (2019). Intra-operative analgesia with remifentanil vs. dexmedetomidine: a systematic review and meta-analysis with trial sequential analysis. Anaesthesia.

[34] Panzer O, Moitra V, Sladen RN (2009). Pharmacology of sedative-analgesic agents: dexmedetomidine, remifentanil, ketamine, volatile anesthetics, and the role of peripheral mu antagonists. Crit Care Clin.

[35] Yoshitomi T, Kohjitani A, Maeda S, Higuchi H, Shimada M, Miyawaki T (2008). Dexmedetomidine enhances the local anesthetic action of lidocaine via an alpha-2A adrenoceptor. Anesth Analg.

[36] Bakan M, Umutoglu T, Topuz U, Uysal H, Bayram M, Kadioglu H, Salihoglu Z (2015). Opioid-free total intravenous anesthesia with propofol, dexmedetomidine and lidocaine infusions for laparoscopic cholecystectomy: a prospective, randomized, double-blinded study. Braz J Anesthesiol.

[37] Xu S-Q, Li Y-H, Wang S-B, Hu SH, Ju X, Xiao JB (2017). Effects of intravenous lidocaine, dexmedetomidine and their combination on postoperative pain and bowel function recovery after abdominal hysterectomy. Minerva Anestesiol.

[38] Brinck ECV, Tiippana E, Heesen M, Bell RF, Straube S, Moore RA, et al. Perioperative intravenous ketamine for acute postoperative pain in adults. Cochrane Database Syst Rev. 2018;12(12):CD012033. 10.1002/14651858.CD012033.pub4. 10.1002/14651858.CD012033.pub4 PMC6360925 30570761

[39] Lahtinen P, Kokki H, Hynynen M, et al. S(+)- ketamine as an analgesic adjunct reduces opioid consumption after cardiac surgery. Anesth Analg. 2004:1295–301. 10.1213/01.ANE.0000133913.07342.B9. 10.1213/01.ANE.0000133913.07342.B9 15502020

[40] Hudetz JA, Patterson KM, Iqbal Z, Gandhi SD, Byrne AJ, Hudetz AG, Warltier DC, Pagel PS (2009). Ketamine attenuates delirium after cardiac surgery with cardiopulmonary bypass. J Cardiothorac Vasc Anesth.

[41] De Oliveira GSJ, Castro-Alves LJ, Khan JH (2013). Perioperative systemic magnesium to minimize postoperative pain: a meta-analysis of randomized controlled trials. Anesthesiology.

[42] Ferasatkish R, Dabbagh A, Alavi M (2008). Effect of magnesium sulfate on extubation time and acute pain in coronary artery bypass surgery. Acta Anaesthesiol Scand.

[43] Cai X, Zhang P, Lu S, Zhang Z, Yu A, Liu D, Wu S (2016). Effects of intraoperative dexmedetomidine on postoperative pain in highly nicotine-dependent patients after thoracic surgery. Medicine (Baltimore).

[44] Greenberg JW, Lancaster TS, Schuessler RB, Melby SJ (2017). Postoperative atrial fibrillation following cardiac surgery: a persistent complication. Eur J Cardio-Thoracic Surg.

[45] Fairley JL, Zhang L, Glassford NJ, Bellomo R (2017). Magnesium status and magnesium therapy in cardiac surgery: a systematic review and meta-analysis focusing on arrhythmia prevention. J Crit Care.

[46] Hu Y-F, Chen Y-J, Lin Y-J, Chen SA (2015). Inflammation and the pathogenesis of atrial fibrillation. Nat Rev Cardiol.

[47] Ueki M, Kawasaki T, Habe K, Hamada K, Kawasaki C, Sata T (2014). The effects of dexmedetomidine on inflammatory mediators after cardiopulmonary bypass. Anaesthesia.

[48] Hayashi Y, Sumikawa K, Maze M, Yamatodani A, Kamibayashi T, Kuro M, Yoshiya I (1991). Dexmedetomidine prevents epinephrine-induced arrhythmias through stimulation of central alpha 2 adrenoceptors in halothane-anesthetized dogs. Anesthesiology.

[49] Fiore AC, Naunheim KS, Taub J, Braun P, McBride LR, Pennington DG, Kaiser GC, Willman VL, Barner HB (1990). Myocardial preservation using lidocaine blood cardioplegia. Ann Thorac Surg.

[50] Fleming IO, Garratt C, Guha R, Desai J, Chaubey S, Wang Y, Leonard S, Kunst G (2016). Aggregation of marginal gains in cardiac surgery: feasibility of a perioperative care bundle for enhanced recovery in cardiac surgical patients. J Cardiothorac Vasc Anesth.

[51] Klinger RY, Cooter M, Berger M (2016). Effect of intravenous lidocaine on the transcerebral inflammatory response during cardiac surgery: a randomized-controlled trial. Can J Anesth.

[52] Santonocito C, Noto A, Crimi C, Sanfilippo F (2018). Remifentanil-induced postoperative hyperalgesia: current perspectives on mechanisms and therapeutic strategies. Local Reg Anesth.

