# Supplementary material for: Feasibility and postoperative opioid sparing effect of an opioid-free anaesthesia in adult cardiac surgery: a retrospective study
Source: BMC Anesthesiol. 2021 Jun 3;21:166. doi: 10.1186/s12871-021-01362-1 (PMC8173983; doi:10.1186/s12871-021-01362-1)
Supplement: Supplementary file 1 — Additional file 1: Table S1. Anesthesia management in OFA (opioid free anesthesia (OFA) and opioid based anesthesia (OBA) groups [file 12871_2021_1362_MOESM1_ESM.docx]

**Table 1.** Anesthesia management in OFA (opioid free anesthesia (OFA) and opioid based anesthesia (OBA) groups

|  | OFA | OBA |
| --- | --- | --- |
| Pre induction given over 15 min | Dexmedetomidine 0.5µg.kg^-1^  Ketamine 0.3mg.kg^-1^  Lidocaine 1.5mg.kg^-1^  MgSO4 3g  Dexamethasone 8mg |  |
| Induction |  | Remifentanil TCI 3.0-10ng.mL^-1^  At the discretion of the anesthesiologist :  Ketamine IV 0.3mg.kg^-1^  Lidocaine IV 1.5mg.kg^-1^  MgSO4 IV 3g  Dexamethasone IV 8 mg |
|  | Propofol TCI 2.0-4.0 µg.mL^-1^  Cisatracurium IV 0.15mg.kg^-1^ | |
| Maintenance | Dexmedetomidine 0.1-0.5µg.kg^-1^.h^-1^  Ketamine 0.25mg.kg^-1^.h^-1^  Lidocaine 1.5mg.kg^-1^.h^-1^ | Remifentanil TCI 3.0-10ng.mL^-1^  At the discretion of the anesthesiologist :  Ketamine 0.25mg.kg^-1^.h^-1^  Lidocaine 1.5mg.kg^-1^.h^-1^ |
|  | Propofol TCI for BIS value 40-60  Cisatracurium 0.1mg.kg^-1^.h^-1^ until aortic unclamping | |
| Intra operative analgesia | Paracetamol 1g and Nefopam 20mg  At the discretion of the anesthesiologist : Ketoprofen 50-100mg | |
|  |  | Morphine 0.1-0.15mg.kg^-1^ |
| End surgery (undressing) | Continuous propofol infusion  Stop dexmedetomidine, remifentanil, lidocaine, ketamine infusion | |

TCI: target-controlled infusion, BIS: bispectral index
